# Earth-mass planets with He atmospheres in the habitable zone of Sun-like stars

**DOI:** 10.1038/s41550-025-02550-6

**Published:** 2025-06-12

**Authors:** Helmut Lammer, Manuel Scherf, Nikolai V. Erkaev, Daria Kubyshkina, Kseniia D. Gorbunova, Luca Fossati, Peter Woitke

**Affiliations:** 1 Space Research Institute, Austrian Academy of Sciences, Graz, Austria; 2 Institute of Computational Modelling, Siberian Branch of the Russian Academy of Sciences, Krasnoyarsk, Russian Federation; 3 Space Research and Planetology, Physics Institute, University of Bern, Bern, Switzerland

**Keywords:** Exoplanets, Computational science

## Abstract

The discovery of many low-mass exoplanets, including several planets within the habitable zone of their host stars, has led to the question of which kind of atmosphere surrounds them. Recent exoplanet detections have revealed the existence of a large population of low-mass planets (<3 *M*_⊕_) with H_2_-dominated atmospheres that must have been accreted from the protoplanetary disk. As the gas disk usually has an ~10% fraction of helium, we model the possible enrichment of the primordial He fraction in the atmosphere of planets with mass between 0.75 *M*_⊕_ and 3.0 *M*_⊕_ that orbit in the classical habitable zone of Sun-like stars. Depending on the mass accreted by the planet during the gas disk phase and the stellar high-energy flux between ~10 and 120 nm, we find that Earth-like planets with masses between ~0.95 *M*_⊕_ and 1.25 *M*_⊕_ inside the habitable zone of Sun-like stars can end up with He-dominated primordial atmospheres. This finding has important implications for the evolution of Earth-like habitats, as these thick helium-enriched primordial atmospheres can inhibit the habitability of these planets. The upcoming generation of giant telescopes, such as the Extremely Large Telescope, may enable us to observe and explore these atmospheres.

## Abstract (web summary)

Simulations of long-term atmospheric evolution show that rocky planets with Earth-like masses in the classical habitable zone of Sun-like stars can retain thick He-dominated primordial atmospheres.

## Main

Observations of protoplanetary disks indicate that there can be several hundred Earth masses of pebbles embedded in each gas disk^1^. These pebbles vanish after a few million years, probably due to radial drift and planetesimal formation outside gas gaps that are produced by growing protoplanets^2^. Mutual collisions between solid matter lead to growth that results in the formation of planetesimals that are kilometres to hundreds of kilometres in size. Through runaway accretion, these produce larger planetary embryos with Moon to Mars masses. Some of them accrete further though collisions to become terrestrial or larger planets^3,4^. The discovery of many highly irradiated low-mass planets indicates that, even for planets close to their host stars, there is a wide diversity in terms of the presence or absence of a primordial H_2_/He-dominated atmosphere. It has been suggested that even planets with masses as low as <2 *M*_⊕_ can have massive primordial atmospheres^5–7^.

Protoplanetary disks are initially composed of ~99% H_2_ and He gas and ~1% solids by mass^8,9^. Atomic hydrogen is four times lighter than He, and thus, depending on the planetary mass, orbital location and stellar extreme ultraviolet (EUV) flux, it escapes more easily, which can result in He atmospheric enrichment and, eventually, in the predominance of primordial He. Indeed, studies and observations of warm (sub-)Neptunes indicate that their atmospheres may be enriched with He or even dominated by He (refs. ^10,11^). The non-detection of He (ref. ^12^) in the atmospheres of the low-mass planets TRAPPIST-1b (*M*_pl_ ≈ 1.3 *M*_⊕_), TRAPPIST-1e (*M*_pl_ ≈ 0.7 *M*_⊕_) and TRAPPIST-1f (*M*_pl_ ≈ 1.3 *M*_⊕_) can be explained by the loss of their atmospheres through thermal^13^ and non-thermal escape processes^14^ induced by the high EUV flux and dense stellar wind exposure over more than 7 Gyr (ref. ^15^). Note that the EUV flux at the orbits of these planets is, at present, up to about 100 times higher compared to that at Earth’s orbit today^16–18^. H_2_/He-dominated atmospheres would still not be stable on Earth-mass planets in the habitable zone (HZ) of the TRAPPIST-1 system, as our simulations below show.

In addition, a primordial atmosphere can be retained only when the accreting protoplanet reaches a certain mass within the lifetime of the gas disk, as the accreted atmosphere would otherwise not be stable and would immediately be lost after nebula dissipation through ‘boil-off’^7^. The discovery of low-mass rocky exoplanets within HZs that still have accreted H_2_/He-dominated primordial atmospheres would, therefore, shed some light on the accretion speed, the formation timescales and the stellar EUV flux evolution.

The lifetimes of inner protoplanetary disks have been measured by infrared (about 1–8 μm) photometry surveys of nearby star-forming regions, such as with the Spitzer Space Telescope^19,20^. For each cluster, the disk frequency is determined as the number of stars with an infrared excess divided by the total number of observed stars. When plotted against cluster age, an exponential dependency is revealed, which can be fitted by exp(−*t*_1/2_/*τ*_a_), where *τ*_a_ is the disk lifetime and *t*_1/2_ = ln(2)*τ*_a_ is the disk half-lifetime. The first study^21^ reported that *t*_1/2_ ≤ 3 Myr (*τ*_a_ ≤ 4.3 Myr). Later measurements found that *τ*_a_ ≈ 2.5 Myr, although slightly longer for low-mass stars and brown dwarfs^19^. The biggest uncertainties in these measurements are the cluster ages^20^, which are determined from the X-ray properties of their stars in combination with pre-main-star evolutionary tracks^22^. Disk half-lifetimes of *t*_1/2_ ≈ 1.3–2 Myr (*τ*_a_ = 1.9–2.9 Myr) are derived when non-magnetic pre-main-star tracks are used, but *t*_1/2_ ≈ 3.5 Myr (*τ*_a_ ≈ 5 Myr) when magnetic pre-main-star models are used^14^. However, the sample selection in these infrared surveys was recently criticized, because the more distant clusters (>200 pc) must be intrinsically brighter to be detected. Hence, they are, on average, younger and denser and have more massive stars, which all leads to shorter median disk lifetimes^16^. Leaving out these distant clusters results in disk lifetimes of *τ*_a_ ≈ 5–10 Myr (ref. ^23^). Using a different method that measures the frequency of accreting stars among the clusters, as indicated by an Hα line in emission detected with the visible multi-objects spectrograph on the Very Large Telescope, then the fraction of accreting stars decreases from ~60% at 1.5–2 Myr to ~2% at 10 Myr, consistent with *τ*_a_ ≈ 2.5–3.5 Myr (ref. ^24^).

Physically, disk dispersal is caused by a combination of (1) accretion onto the star, (2) mass loss in the form of EUV photoevaporation disk winds^25,26^, (3) thermo-magnetic disk winds^27^ and (4) accretion onto the forming planets. Both types of disk winds are triggered by the EUV irradiation from the central star, which ionizes and heats the disk surface in the inner regions. In dense clusters, however, the external EUV irradiation from nearby massive stars is expected to generate further winds launched from the outer disk regions, resulting in shorter overall disk lifetimes. Substantially lower lifetimes (~1 Myr instead of ~3 Myr) have been reported for massive star-forming regions that host O-stars (for example, Fig. 13 in ref. ^28^). In addition, low metallicities also seem to shorten the disk lifetimes^22,29^, although the statistical evidence for this effect is poor, as most of the observable nearby star-forming region have similar metallicities. If EUV photoevaporation winds are effective, then they can cause a sudden and complete local dispersal of the gas in the wind-launching zones of the disks^18,25^, an effect that is not covered by the observable overall disk lifetime. Rocky planets forming in such zones might experience a sudden loss of their gaseous environment, which is discussed for the peculiar evolution of the young, hot Neptune AU Mic b by ref. ^30^ and is possibly related to the formation of the hot-Neptune desert^31^.

The outcome depends on the accreted planetary rocky mass fraction at the end of the protoplanetary nebula and on the incident stellar EUV flux. For planets in the HZ of solar-like stars, the accumulated primordial atmospheres are lost within a hundred thousand to several million years, if the rocky part of the planet remains below ~0.75 *M*_⊕_ (refs. ^32–34^). More massive planets, however, will not lose their accumulated primordial atmosphere. If they do, this process may take several hundred million years^7,35^.

We consider several different hypothetical planets with accreted rocky masses *M*_pl_ between 0.75 *M*_⊕_ and 3.0 *M*_⊕_. The photospheric radius *r*_ph_ (the atmospheric altitude where the optical depth *τ* = 1, typically at an atmospheric pressure *P*_ph_ ≈ 100 mbar^36,37^) of the specific planet depends on the mass of the H_2_/He-dominated primordial atmosphere of the planet and the temperature of the rocky part of the planet, both of which are poorly constrained. During disk dissipation, the planet cools and its photospheric radius shrinks through thermal escape driven by boil-off^34–37^ until the planet becomes compact enough for EUV-driven hydrodynamic escape to become the dominant atmospheric escape mechanism^34,36,37^.

Note that compared to EUV radiation, X-rays^38^ contribute to thermal escape only at very young stellar ages during a very short period when X-ray and EUV luminosities are comparable in magnitude. However, X-rays are negligible over evolutionary timescales^39–41^ in the HZ, as studied in our cases. Early approaches were very much simplified^38^ and did not consider that X-ray photons are more likely to excite an atom than to ionize it^42^. In addition, X-rays contribute to heating only in very narrow atmospheric layers^41–43^. Because of this, we applied our upper-atmosphere multispecies hydrodynamic model only to the evolving EUV radiation.

The amount of the primordial atmosphere remaining after the boil-off phase and, thus, the post-boil-off photospheric radius for low-mass planets are roughly independent of the pre-boil-off conditions^36,44^ and can be characterized by the equilibrium temperature *T*_0_, which is related to the orbital location, and by the planetary mass *M*_pl_. Therefore, we started our evolution simulations after the end of the boil-off phase and adopted the post-boil-off photospheric radius as the initial radius *r*_ph,in_. As the duration of the boil-off phase is short (typically less than a million years for low-mass planets^44,45^), we also assumed that its conclusion coincides with the time of the protoplanetary disk dispersal. This age was the starting time of our simulations.

To estimate the values of *r*_ph,in_ for each of the considered *M*_pl_, we performed a series of hydrodynamic simulations^36^ adopting the stellar parameters corresponding to the starting time (but not yet considering the evolution) and different *r*_ph_ values between 1 and 4.5 Earth radii (with a step of 0.1 Earth radii and exact range depending on the specific *M*_pl_). We then compared the atmospheric mass-loss rates predicted by the hydrodynamic model (accounting for both EUV-driven and boil-off escape mechanisms) to the prediction of the energy-limited approximations (accounting for EUV-driven escape only) and adopted the photospheric radius where the two mass-loss rates were equal as the initial radius for the evolution simulations *r*_ph,in_.

Afterward, for each pair of considered *M*_pl_ and *r*_ph,in_ values, we modelled the evolution of the model planet. The evolution of the stellar EUV luminosity is given by the power laws for G-type stars that are slow, moderate or fast rotators, as described in Methods. The equilibrium temperature *T*_0_ at 1 au was assumed to be 250 K for all scenarios studied. The mass fraction *f* of the mass of the H_2_/He-dominated atmosphere *M*_at_ to the mass of the rocky part *M*_pl_ depends on the photospheric radius *r*_ph_ as^34,37^

1 $$f=\frac{{M}_{\rm{at}}}{{M}_{\rm{pl}}}={\left\{\frac{{\log}_{10}(r_{{{\mathrm{ph}}}})+0.07151}{1.14767}\right\}}^{3.12178}.$$

The mass fraction *f* evolves with time due to the EUV-driven hydrodynamic escape of hydrogen and He, which reduces *M*_at_ along with the evolving stellar EUV luminosity. Table 1 lists examples of the initial atmospheric mass fractions *f*_in_ obtained following equation (1) for each considered planetary mass at the beginning of the evolution. Although the determination of *f*_in_ considers the luminosity of the cooling rocky core when estimating the photospheric radius, note that the obtained atmospheric mass fractions in Table 1 represent average cases. In reality, the mass of the accreted atmosphere could deviate from the calculated value due to, for example, differences in the opacity of the accreting gas^46,47^ or giant impacts that may also modify the initial mass of the captured primordial atmosphere^48,49^. However, if a planet accretes more atmosphere than calculated with equation (1), any extra atmospheric mass above the calculated threshold will be almost immediately lost through boil-off as the extra gas may not be gravitationally stable. If, on the other hand, a planet accretes less atmosphere than expected from equation (1), for example, due to the disk being very gas-poor or atmospheric erosion through giant impacts, the final planetary mass needed to obtain a He-dominated atmosphere could be slightly higher than obtained from our model. We also remark that for all considered planets, the atmospheric mass *M*_at_ was substantially smaller than the mass of the rocky body, so that its contribution to the total planetary mass was negligible. For each of our cases, we assumed that the primordial atmosphere contains an initial abundance ratio of 90% (H_2_) to 10% (He).

**Table 1:** Examples^a^ of normalized planetary masses *M*_pl_ (*M*_⊕_) and the corresponding initial atmospheric mass fraction *f*_in_ obtained from equation (1) following the boil-off phase

Case	1	2	3	4	5	6	7	8	9	10	11	12	13
$\frac{{M}_{\rm{pl}}}{{M}_{\oplus }}$	0.75	0.85	0.9	0.95	0.97	1.0	1.02	1.06	1.25	1.3	1.5	2.0	3.0
*r*_ph_	1.22	1.36	1.40	1.46	1.48	1.72	1.75	1.82	2.02	2.04	2.09	2.66	3.82
*f*_in_	0.002	0.0046	0.0061	0.0073	0.01	0.016	0.018	0.02	0.03	0.032	0.035	0.073	0.17

^a^Simulations were performed with a smaller grid of planetary masses than displayed in this table.

We assumed an average disk lifetime of 5 Myr, but our results were independent of this choice because, at these young ages, the EUV emission of solar-like stars is in the saturation regime regardless of their rotation rate^37,50^. From 5 Myr onwards, we evolved the atmosphere of each of the model planets listed in Table 1 using three different EUV flux evolution power laws. To model the mass-loss evolution of the H_2_/He-dominated primordial atmosphere and the fractionation of He compared to hydrogen, we applied the multispecies hydrodynamic upper-atmosphere model of Erkaev et al.^37^, which is described in Methods.

At each time step, the model produced snapshots of mass-loss rates of the escaping primordial atmosphere, the evolving atmospheric mass fraction and the changing atmospheric H_2_ and He ratio. The change in the photospheric radius *r*_ph_ over time was calculated with equation (1) based on the atmospheric mass fraction *f* obtained after each step of the evolution. During our calculations, we made sure that the escaping primordial atmosphere was in the collisional regime. The simulation stopped when the atmospheric pressure reached <100 mbar or once hydrogen loss had become negligible because the escape switched from the EUV-driven hydrodynamic escape regime to Jean’s escape.

**Fig. 1:**
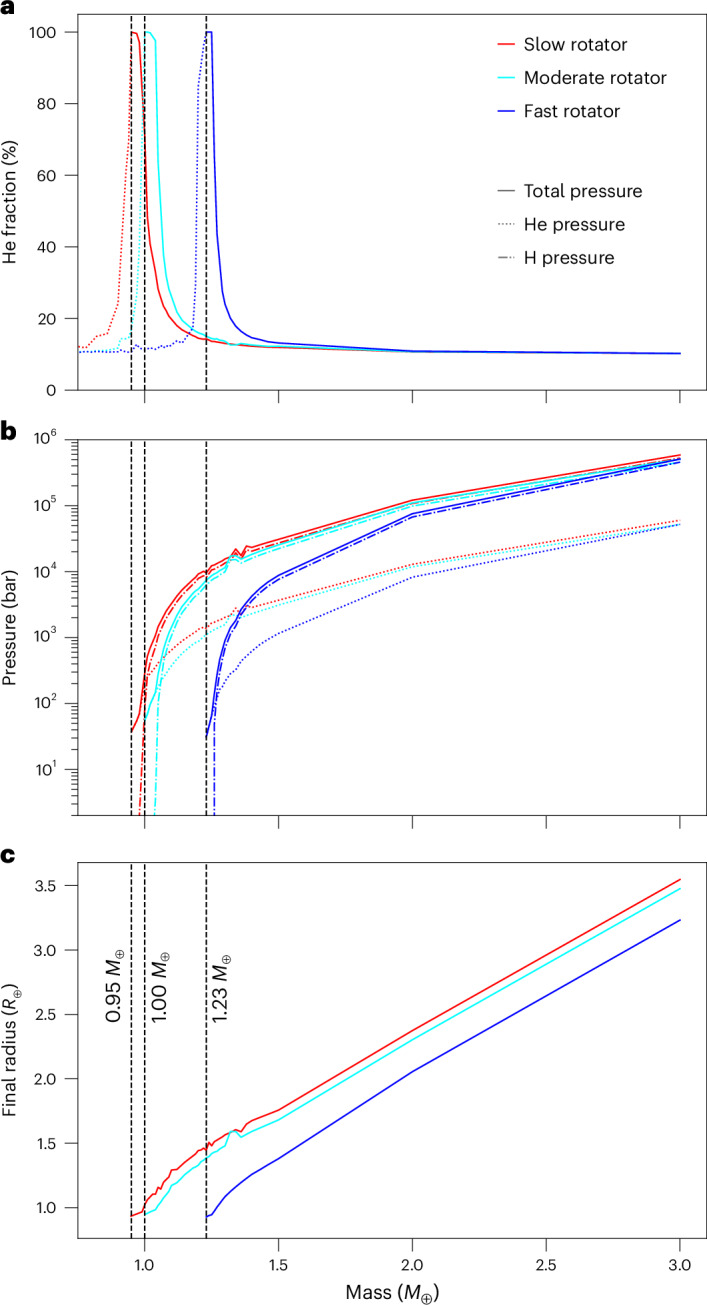
Atmospheric He fractions, pressures and optical radii. **a**, Atmospheric He fraction at the end of the evolution simulations as a function of planetary mass and considering the solar-like host star as a slow-, moderate- or fast-rotating young G-type star. Planets in the dotted part of the lines subsequently lose their remaining primordial atmospheres in a geologically short timescale. Planets in the solid parts keep remnants of their thermally stable primordial atmospheres. The vertical black dashed lines show the critical planetary mass above which thermally stable atmospheres remain. **b**, The total and partial pressures for planets with thermally stable atmospheres. **c**, The final broadband photospheric radii *r*_ph_ (in the optical) of the planets with thermally stable atmospheres. Source data

Figure 1a shows the atomic He fraction in the primordial atmosphere as a function of planetary mass in the range 0.75–3.0 *M*_⊕_, as obtained at the end of our simulations for solar-like stars that are slow, moderate or fast rotators. Each planet started with a He fraction of 10% in the atmosphere with the rest being H. If hydrodynamic escape is strong, both particles escape with similar fluxes, so that the ratio between He and H does not change. If hydrogen is lost faster than helium, the He also finally escapes, so that the whole primordial atmosphere is lost. The cases where the primordial atmosphere is eventually lost are shown with dotted lines. However, for certain planetary masses, the protoplanet becomes so massive that He stops escaping efficiently, whereas the hydrogen still escapes strongly. The solid lines correspond to cases where the primordial atmospheres remain. One can see peaks for 0.95 *M*_⊕_, 1.0 *M*_⊕_ and 1.25 *M*_⊕_ planets that end with ~100% He atmospheres, after these planets have been exposed to slow-, moderate- or fast-rotating young G-stars for ~1.28, ~2.36 and ~2.37 Gyr, respectively. These peaks shifted to higher planetary masses for stars that are more active. After the peaks, the gravity of the protoplanets becomes so strong that, at some point during their atmospheric evolution, neither H nor He can escape efficiently. For these cases, the fraction of He remains relatively constant in the atmosphere and the planets will keep substantial hydrogen/He-dominated atmospheres with almost the same He fraction as at the start of the simulation. Figure 1b,c shows the corresponding partial pressures of the remaining hydrogen and He atmospheres and the final photospheric radii in units of *R*_⊕_. For lower-mass planets, the entire primordial atmosphere eventually escapes, hydrogen as well as He.

Also note that, by reproducing the present atmospheric ^36^Ar/^38^Ar, ^20^Ne/^22^Ne, ^36^Ar/^22^Ne, isotope and bulk K/U ratios, early Earth’s evolution can be explained if proto-Earth had accreted masses between ~0.53 *M*_⊕_ and ~0.58 *M*_⊕_ by the time the nebula gas dissipated^34^. From the results shown in Fig. 1 and depending on the initial rotation rate of the Sun, one can see that if the proto-Earth had accreted to its final mass within the solar nebula, it would not have lost a large fraction of the accreted primordial atmosphere, so that the Earth would not have developed into a planet suitable for life as we know it.

**Fig. 2:**
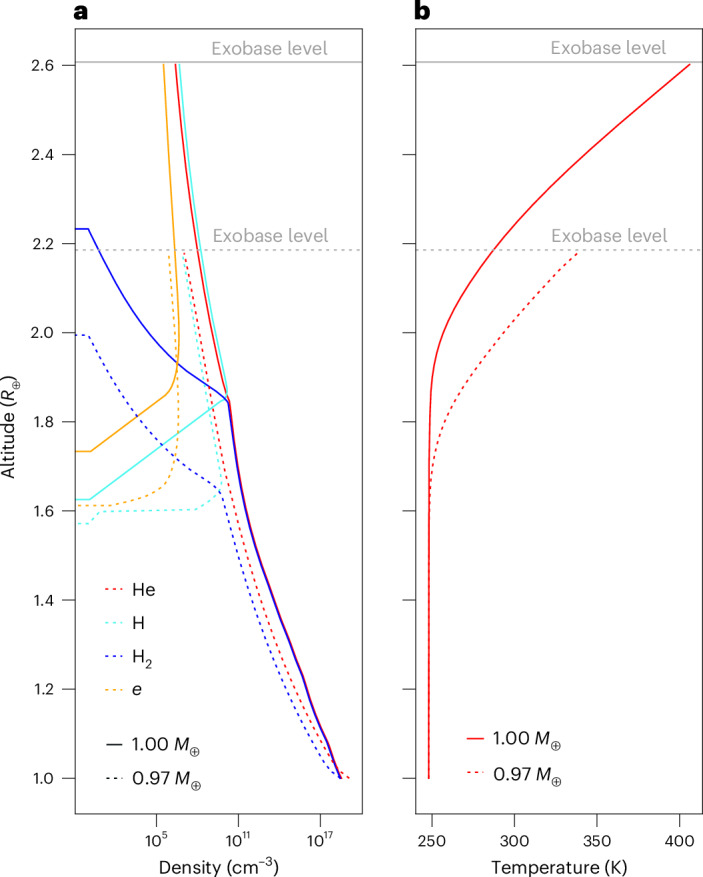
Examples of atmospheric profiles. **a**,**b**, Atmospheric number density (**a**) and temperature (**b**) profiles for 1.0 *M*_⊕_ (solid lines) and 0.97 *M*_⊕_ (dotted lines) HZ planets slowly rotating around and exposed to the EUV flux evolution of a solar-like young star. The plots show the last time step of our simulation. For 0.97 *M*_⊕_, hydrogen is subsequently completely lost. The yellow lines show the electron number density in the upper atmosphere. Source data

Figure 2a shows the atmospheric density profiles for He and H_2_ and the dissociation product H as a function of distance in units of *R*_⊕_, one simulation step before the hydrogen abundance is mainly lost from the planet. As mentioned above, we stopped our simulation when the hydrogen partial pressure ≤100 mbar. The profiles correspond to a simulation with a slow-rotating young solar-like G-type star. One can see that the H_2_ molecules dissociated above ~1.6 *R*_⊕_. Figure 2b shows the corresponding temperature profiles and exobase levels.

**Fig. 3:**
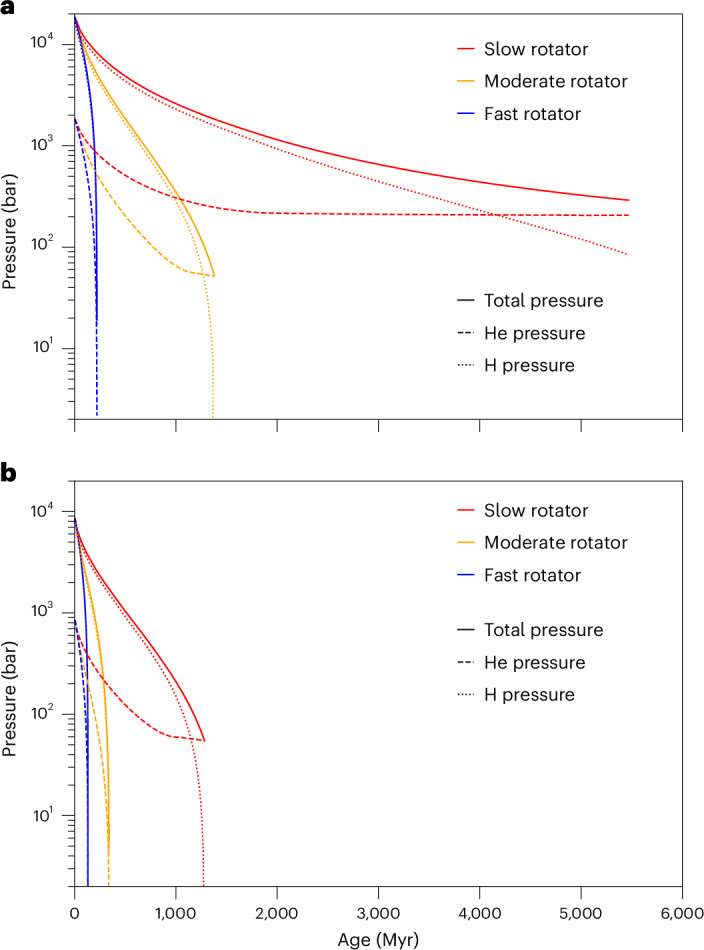
Atmospheric pressure evolution. **a**, Evolution of the total (solid lines) and surface partial pressures of hydrogen (dotted lines) and He (dashed lines) for an Earth-mass HZ planet around a solar-like star exposed to the EUV flux evolution of a slow (red), moderate (green) or fast (blue) rotator. **b**, The same for a planet of mass 0.97 *M*_⊕_. Source data

Figure 3 shows the corresponding total surface pressure of the primordial atmosphere and the partial surface pressures of hydrogen and He for a planet of mass 1.0 *M*_⊕_ (Fig. 3a) and with 0.97 *M*_⊕_ (Fig. 3b) that is exposed to the EUV flux of a slow-, moderate- or fast-rotating young solar-like host star. For a fast rotator, the accreted primordial atmosphere of a planet of mass 1.0 *M*_⊕_ is lost within ~220 Myr. For a moderate rotator, ~50 bar of He remains after ~1.4 Gyr with an atmospheric He fraction *f*_He_ = 97%. By contrast, a substantial primordial atmosphere remains for a slow rotator with *f*_He_ = 71%. At planetary masses of ~0.95–0.97 *M*_⊕_, ~1.0–1.02 *M*_⊕_ and ~1.23–1.25 *M*_⊕_, which correspond to the peaks in the atmospheric He fraction in our simulations, 55, 90 and 51 bar of He remain at the planets, respectively, thereby forming substantial He-dominated primordial atmospheres.

Note that the power laws considered for the EUV flux evolution of slow and fast rotators represent minimum and maximum pathways for the EUV flux evolution. It is, therefore, probable that planets in the HZ of solar-like stars can be found with He-dominated or strongly He-enriched primordial atmospheres for any planetary mass between ~0.95 *M*_⊕_ and ~1.25 *M*_⊕_, depending on the specific stellar EUV flux evolution scenario. For later stellar spectral types, we expect that atmospheric He enrichment will shift to planets with higher masses, such as (sub-)Neptunes^10,11^, because lower-mass stars can remain active for much longer than G-type stars.

Similarly, if one considers planets in close orbit around their host stars (<0.1 au), one would expect to find planets with He-dominated atmospheres within the warm to hot (sub-)Neptune population^10^. The strong fractionation of He and deuterium was recently studied and is expected in sub-Neptune atmospheres along the radius valley^11^. This theoretical prediction is supported by the discovery of extended and escaping He atmospheres, like those detected for the young mini-Neptune TOI 560.01 (ref. ^51^), the warm Neptune-mass planet HAT-P-11b (ref. ^52^) and the hot Saturn-mass planet WASP-69 b (ref. ^53^).

We studied the possible detectability of the metastable He i triplet at 1,083 nm in the atmosphere of an HZ Earth-mass planet orbiting at 1 au around a Sun-like star considering an atmospheric fraction of 30%, 50% or 70% He. Thus, we used the atmospheric structures obtained from the hydrodynamic simulations as input to radiative transfer modelling under non-local thermodynamical equilibrium conducted with Cloudy^54,55^ through the Cloudy for Exoplanets interface^56,57^, for which the predicted metastable He i absorption strength has been validated against literature values for the hot Jupiter HD 209458 b (ref. ^57^).

**Fig. 4:**
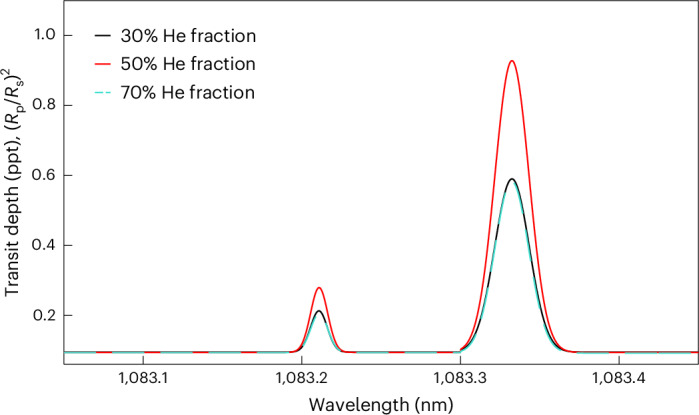
Transmission spectra of the He i metastable triplet. The transmission spectra are for an Earth-mass planet with an atmospheric He enrichment of 30% (black), 50% (red) and 70% (blue) orbiting in the HZ of a Sun-like star. Source data

Figure 4 shows the He i triplet transmission spectra computed for the three considered cases at a spectral resolution of 100,000. The He i absorption strength increased on going from 30% to 50% enrichment, but it then decreased at 70% enrichment as a result of the higher mean molecular weight and, thus, shorter pressure scale height (atmospheric size).

Among the three cases, we obtained the strongest absorption for the 50% He enrichment case, for which the transit radius in the He i metastable triplet could increase by up to a factor of ~3 compared to a He-free atmosphere. However, the transmission signal is a factor of a few smaller than what can be detected with current instrumentation^58–60^. However, the Extremely Large Telescope could soon enable us to explore how ubiquitous He enrichment is in the atmospheres of low-mass planets.

From the results of this study and the before-mentioned observational evidence of He extended and escaping atmospheres on (sub-)Neptunes to sub-Jupiters, one could expect the possible existence of a population of terrestrial HZ planets hosting He-dominated primordial atmospheres and partial surface pressures from a few bars to hundreds of bars. The future discovery of such planets will not only advance our understanding of the accretion speed of terrestrial exoplanets but will also open questions related to the habitability of Earth-like planets and whether such He-dominated atmospheres are dangerous for hypothetical life forms, especially complex aerobic life as we know it. Little is known about how life forms are affected by large atmospheric He fractions, but the low mixing ratio of oxygen expected for a He-dominated primordial atmosphere can hardly sustain aerobic life. For example, it is known that if He fills the lungs of mammals, it produces a diffusion gradient that washes out the O_2_ stored in the blood, dropping O_2_ to a lethal level within seconds^61^. One can conclude that thoroughly understanding the complex interplay between the accretion speed of a planet, the related lifetime of the gas disk, the accumulation of primordial atmospheres and the EUV flux evolution of the host star is key to understanding how planets can develop into Earth-like habitats that could, indeed, evolve N_2_/O_2_-dominated secondary atmospheres.

## Methods

### EUV flux of young G-type stars and the corresponding power laws

The EUV flux of the host star becomes the driving force of the hydrodynamic escape of primordial atmospheres as soon as boil-off stops (which is after the disk evaporates). During the early phase of the stellar evolution (~1 Gyr), the stellar EUV flux correlates with the rotation rate^50^. From X-ray observations collected with the ROSAT, XMM-Newton and Chandra satellites, we know that Sun-like stars born as slow, moderate or fast rotators experience an X-ray and EUV saturation phase *t*_sat_ that lasts ~5, ~25 or ~225 Myr, respectively^31^. As input to the multispecies hydrodynamic upper-atmosphere model, we applied the same power laws that describe the evolution of the EUV luminosity ${L}_\mathrm{K}^\mathrm{s,m,f}$ of slow (s), moderate (m) and fast (f) rotators within the wavelength range 10–120 nm as given in (refs. ^28,31^):

2 $${L}_{\rm{EUV}}^\mathrm{s}=5.75\times {10}^{31}{t}^{\,-0.93}\,{\rm{erg}}\,{{\rm{s}}}^{-1},\qquad{}t\ge{}t_{{\rm{sat}}}=5\,{\rm{Myr}},$$

3 $${L}_{\rm{EUV}}^\mathrm{m}=4.7\times {10}^{32}{t}^{\,-1.18}\,{\rm{erg}}\,{{\rm{s}}}^{-1},\qquad{t}\ge {t}_{{\rm{sat}}}=25\,{\rm{Myr}},$$

4 $${L}_{\rm{EUV}}^\mathrm{f}=1.2\times {10}^{36}{t}^{\,-2.15}\,{\rm{erg}}\,{{\rm{s}}}^{-1},\qquad{t}\ge {t}_{{\rm{sat}}}=225\,{\rm{Myr}}.$$

The EUV surface flux of the slow, moderate and fast rotators for the planets in Table 1 can then be written as

5 $${I}_{\rm{EUV}}^{\,{\mathrm{s,m,f}}}=\frac{{L}_{\rm{EUV}}^{\mathrm{s,m,f}}}{4\uppi {d}^{2}}\,{\rm{erg}}\,{{\rm{cm}}}^{-2}\,{{\rm{s}}}^{-1},$$

with the orbital distance *d* inside the HZ at 1 au. ${I}_{\rm{EUV}}^{i}$ can also be seen as the EUV intensity or energy received per unit area in a given time outside the atmosphere at the orbit of the planet^37^.

### Multispecies hydrodynamic upper-atmosphere model

The details of the applied hydrodynamic upper-atmosphere model used for the H-dragging of the He atoms, including the numerical algorithm and specific parameter values (for example, dissociation rates, ionization rates and heating efficiency) can be found in Erkaev et al.^37^. Here, we describe only the main mathematical framework and numerical algorithms that we applied to model the evolution of the hydrogen and He contents of the primordial atmospheres over time (for example, mass loss and evolutionary change of the H_2_/He ratio). We applied the following system of equations for the mass conservation of H, H^+^, H_2_, H_2_^+^ and He:

6 $$\frac{\partial {n}_\mathrm{H}}{\partial t}+\frac{1}{{r}^{2}}\frac{\partial \left({n}_\mathrm{H}V{r}^{2}\right)}{\partial t}=-{\nu }_\mathrm{H}{n}_\mathrm{H}+{\alpha }_\mathrm{H}{r}_{e}{n}_{\rm{H}^+}+2{\alpha }_{\rm{H}_{2}}{n}_{e}{n}_{\rm{H}_{2}^+}-2{\gamma }_\mathrm{H}n{n}_\mathrm{H}^{2},$$

7 $$\frac{\partial {n}_{\rm{H}^+}}{\partial t}+\frac{1}{{r}^{2}}\frac{\partial \left({n}_{\rm{H}^+}V{r}^{2}\right)}{\partial t}={\nu }_\mathrm{H}{n}_\mathrm{H}-{\alpha }_\mathrm{H}{n}_{e}{n}_{\rm{H}^+},$$

8 $$\frac{\partial {n}_{\rm{H}_{2}}}{\partial t}+\frac{1}{{r}^{2}}\frac{\partial \left({n}_{\rm{H}_{2}}V{r}^{2}\right)}{\partial t}=-{\nu }_{\rm{H}_{2}}{n}_{\rm{H}_{2}}+{\gamma }_\mathrm{H}n{n}^{2},$$

9 $$\frac{\partial {n}_{\rm{H}_{2}^+}}{\partial t}+\frac{1}{{r}^{2}}\frac{\partial \left({n}_{\rm{H}_{2}^+}V{r}^{2}\right)}{\partial t}={\nu }_{\rm{H}_{2}}{n}_{\rm{H}_{2}}-{\alpha }_{\rm{H}_{2}}{n}_{e}{n}_{\rm{H}_{2}^+},$$

10 $$\frac{\partial {n}_{\rm{He}}}{\partial t}+\frac{1}{{r}^{2}}\frac{\partial \left({n}_{\rm{He}}{V}_{\rm{He}}{r}^{2}\right)}{\partial t}=0.$$

Here, *V* and *V*_He_ are the velocities of the hydrogen and He particles, *n*_H_, $n_{\rm{H}_2}$, $n_{\rm{H}^+}$, $n_{\rm{H}_2^+}$ are the number densities of the hydrogen atoms, molecules and ions, respectively, *n*_He_ is the helium number density, *n* the total number density, and *n*_*e*_ = ($n_{\rm{H}^+}$ + $n_{\rm{H}_2^+}$) is the electron number density determined by the condition of quasi-neutrality. γ_H_ is the rate of reaction H + H→ H_2_, *α*_H_ is the recombination rate of atomic hydrogen, $\alpha_{\rm{H}_2}$ is the dissociation rate, and *ν*_H_ and $\nu_{\rm{H}_2}$ are the ionization rates of atomic and molecular hydrogen. For these hydrogen constituents, we introduce the total mass density, which is a sum of the partial mass densities:

11 $$\frac{\partial \rho }{\partial t}+\frac{1}{{r}^{2}}\frac{\partial \left(\rho V{r}^{2}\right)}{\partial t}=0.$$

For He, we apply a similar equation:

12 $$\frac{\partial {\rho }_{\rm{He}}}{\partial t}+\frac{1}{{r}^{2}}\frac{\partial \left({\rho }_{\rm{He}}{V}_{\rm{He}}{r}^{2}\right)}{\partial t}=0.$$

We assumed that all species have the same temperature, which is governed by the energy conservation equation:

13 $$\rho \frac{\mathrm{d}E}{\mathrm{d}t}={Q}_{\rm{EUV}}-{W}_{\rm{Ly}\upalpha}+\frac{\partial }{{r}^{2}\partial r}\left({r}^{2}\chi \frac{\partial T}{\partial r}\right)-P\frac{\partial \left({r}^{2}V\right)}{{r}^{2}\partial r},$$

where *E* is the thermal energy and *χ* is the thermal conductivity^62^. *Q*_EUV_ is the EUV volume heating rate in the upper atmosphere, which is proportional to the EUV energy flux absorbed in the upper atmosphere, the ratio of the net local heating rate to the rate of the stellar radiative absorption, and the average EUV photo-absorption cross section of hydrogen atoms and molecules^17^. *W*_Lyα_ is the cooling due to Lyman-α emission, *ρ* is the total mass density of all hydrogen species, *P* is the total pressure of hydrogen and electrons, *T* is the temperature and *k*_B_ is the Boltzmann constant. The velocities are determined by the Euler momentum equations:

14 $$\rho \frac{\mathrm{d}V}{\mathrm{d}t}+\nabla P=g\rho +{\rho }_{\rm{He}}\frac{{m}_\mathrm{H}{\nu }_\mathrm{c}}{{m}_\mathrm{H}+{m}_{\rm{He}}}\left({V}_{\rm{He}}-V\,\right),$$

15 $${\rho }_{\rm{He}}\frac{\mathrm{d}{V}_{\rm{He}}}{\mathrm{d}t}+\nabla {P}_{\rm{He}}=g{\rho }_{\rm{He}}-{\rho }_{\rm{He}}\frac{{m}_\mathrm{H}{\nu }_\mathrm{c}}{{m}_\mathrm{H}+{m}_{\rm{He}}}\left({V}_{\rm{He}}-V\,\right).$$

Here, *g* is the gravitational acceleration, *m*_H_ is the mass of a hydrogen atom, *m*_He_ is the mass of a helium atom, *P*_He_ is the partial pressure of helium and *ν*_c_ is the collision frequency between these two species. For computational convenience, we use the following normalizations:

16 $${X}_{\rm{He}}={\rho }_{\rm{He}}/\rho ,$$

17 $$\widetilde{v}={\nu }_\mathrm{c}/{\nu }_\mathrm{c,{ph}},$$

18 $$\widetilde{V}=V/{V}_{\rm{ph}},$$

19 $${V}_{\rm{ph}}={\left(k{T}_{\rm{ph}}/{m}_\mathrm{H}\right)}^{1/2},$$

20 $$\widetilde{P}=\frac{P}{\left({\rho }_{\rm{ph}}{V}_{\rm{ph}}^{2}\right)},$$

21 $$\widetilde{\rho }=\frac{\rho }{{\rho }_{\rm{ph}}},$$

22 $$u=\frac{\left({V}_{\rm{He}}-V\,\right)}{{V}_{\rm{ph}}},$$

23 $$\widetilde{T}=\frac{T}{{T}_{\rm{ph}}},$$

24 $$\widetilde{m}=\frac{{m}_{\rm{He}}}{{m}_\mathrm{H}},$$

25 $$\widetilde{r}=\frac{r}{{r}_{\rm{ph}}},$$

26 $$\widetilde{t}=\frac{t{V}_{\rm{ph}}}{{r}_{\rm{ph}}},$$

27 $$\varepsilon ={V}_{\rm{ph}}/\left({\nu }_\mathrm{c,{ph}}{r}_{\rm{ph}}\right),$$

28 $$\lambda ={g}_{\rm{ph}}{r}_{\rm{ph}}/\left(k{T}_{\rm{ph}}\right),$$

29 $$\varGamma ={L}_\mathrm{H}/\left({\rho }_{\rm{ph}}{V}_{\rm{ph}}{r}_{\rm{ph}}^{2}\right).$$

Here, *L*_H_ is the loss rate of hydrogen. Subscript ‘ph’ is used for parameters at the lower boundary, which can be equated with the photospheric radius *r*_ph_, which is the specific planetocentric distance and atmospheric level where the atmosphere is opaque below and transparent above for the visible part of the spectrum along the radial distance *r* as long as the particles in the expanding thermosphere experience collisions. Hereafter, we leave out the ‘∼’ above the normalized quantities for simplicity. By neglecting the second-order terms involving the small parameter *ε*, one can derive the equation for the relative velocity *u* = (*V*_He_ *−* *V*) to obtain:

30 $$\rho {X}_{\rm{He}}\left({V}_{\rm{He}}-V\,\right)=\varepsilon \frac{\left(m+1\right)}{\nu \left(1+{X}_{\rm{He}}\right)}\left[\frac{\partial P}{\partial r}{X}_{\rm{He}}-\frac{\partial {P}_{\rm{He}}}{\partial r}\right].$$

Using equation (30) together with the continuity equation (12), one can derive the equation for the He fraction for a stationary flow:

31 $$\rho V{r}^{2}\frac{\partial {X}_{\rm{He}}}{\partial r}+\frac{\partial }{\partial r}\left\{\varepsilon {r}^{2}\frac{\left(m+1\right)}{\nu \left(1+{X}_{\rm{He}}\right)}\left[\frac{\partial P}{\partial r}{X}_{\rm{He}}-\frac{\partial {P}_{\rm{He}}}{\partial r}\right]\right\}=0.$$

with the partial pressure *P*_He_ of the helium constituent:

32 $${P}_{\rm{He}}={X}_{\rm{He}}P\xi /m,$$

33 $$\xi ={(1+{X}_{\rm{H}^+}-0.5{X}_{\rm{H}_{2}})}^{-1},$$

34 $${X}_{\rm{H}^+}={n}_{\rm{H}^+}{m}_\mathrm{H}/\rho ,$$

35 $${X}_{\rm{H}_{2}}=2{n}_{\rm{H}_{2}}{m}_\mathrm{H}/\rho .$$

Integrating this equation and assuming *ρVr*^2^ = *Γ* = const, one gets

36 $$\varGamma \left({X}_{\rm{He}}-{X}_{\rm{He}\infty }\right)+\varepsilon {r}^{2}\frac{\left(m+1\right)}{\nu \left(1+{X}_{\rm{He}}\right)}\left[\frac{\partial P}{\partial r}{X}_{\rm{He}}-\frac{\partial {P}_{\rm{He}}}{\partial r}\right]=0,$$

where X_He∞_ is the He mass fraction at infinity. By integrating this equation analytically, we found that the He fraction depends on the radial distance as

37 $${X}_{\rm{He}}={X}_{\rm{He}\infty }\int_{\psi }^{{\psi }_{\infty }}\exp \left(\psi -\psi^{\prime} \right){\left(1+\varepsilon B\right)}^{-1}\,\mathrm{d}\psi^\prime,$$

38 $$\psi =\frac{1}{\varepsilon }\int_{1}^{r}\left(1+\varepsilon B\right){A}^{-1}\,\mathrm{d}r^\prime,$$

39 $$A=\frac{\left(1+m\right){r}^{2}\xi P}{m\varGamma \nu },$$

40 $$B=\frac{\left(1+m\right){r}^{2}}{m\varGamma \nu }\frac{\mathrm{d}\left[\left(m-\xi\,\right)P\right]}{\mathrm{d}r}.$$

Here, *ψ*′ and *r*′ are the variables the equation integrates. Integrating equations (36)–(39) by parts, one obtains the following expression:

41 $$\begin{array}{l}{X}_{\rm{He}}\\={X}_{\rm{He}\infty }\frac{1}{\left(1+\varepsilon B\right)}\left[1-\left(1+\varepsilon B\right){\varepsilon }^{2}\int_{\psi }^{{\psi }_{\infty }}{\left(1+\varepsilon B\right)}^{-3}\,\frac{\mathrm{d}B}{\mathrm{d}r}A\exp \left(\psi -\psi^{\prime} \right)\,\mathrm{d}\psi^{\prime} \right].\end{array}$$

Neglecting the second-order term proportional to *ε* in this equation, we obtained the analytical formula for the dragging factor *κ*_He_ defined as a ratio *X*_He∞_/*X*_He,ph_:

42 $$\begin{array}{l}{\kappa }_{\rm{He}}={X}_{\rm{He}\infty }/{X}_\mathrm{{He},{ph}}\\ \quad \,\,\,\,\,={X}_\mathrm{{He},{ph}}\left(1+\varepsilon B\right)={X}_\mathrm{{He},{ph}}{\left\{1+\varepsilon \frac{\left(1+m\right)}{m\varGamma }\frac{\rm{d}}{\mathrm{d}r}\left[P\left(m-\xi \right)\right]\right\}}_{\rm{ph}},\end{array}$$

where *X*_He,ph_ is the He mass fraction at the lower boundary, respectively. The variation over time of the hydrogen and He masses in the atmosphere are determined by the mass conservation equations:

43 $$\frac{{\rm{d}}{M}_{\rm{at}}}{{\rm{d}}t}=-{L}_\mathrm{H},$$

44 $$\frac{\mathrm{d}\left({M}_{\rm{at}}{X}_\mathrm{{He},{ph}}\right)}{\mathrm{d}t}=-{L}_\mathrm{H}{\kappa }_{\rm{He}}{X}_\mathrm{{He},{ph}}.$$

where *M*_at_ is the mass of the primordial atmosphere. The last equation can be rearranged into a more convenient form:

45 $$\frac{\mathrm{d}\left({X}_\mathrm{{He},{ph}}\right)}{\mathrm{d}t}=\frac{{L}_\mathrm{H}\left(1-{\kappa }_{\rm{He}}\right)}{{M}_{\rm{at}}}{X}_\mathrm{{He},{ph}}.$$

This indicates clearly that the relative mass fraction of He in the evolving primordial atmosphere is an increasing function of time during the planetary evolution.

## Source data

Source Data Fig. 1 Data points for Fig. 1a–c.

Source Data Fig. 2 Data points for Fig. 2a,b.

Source Data Fig. 3 Data points for Fig. 3a,b.

Source Data Fig. 4 Data points for Fig. 4.

## Data Availability

The data generated by this study and needed to reproduce Figs. 1–4 are free to use under a Creative Commons license CC-BY 4.0 licence and available via Figshare at 10.6084/m9.figshare.28533617 (ref. ^62^). Source data are provided with this paper.

## Code availability

The code is not publicly available. Please contact the corresponding authors to discuss potential collaborations related to the hydrodynamic atmosphere code used in this study.

## References

[1] Lambrechts, M. & Johansen, A. Rapid growth of gas-giant cores by pebble accretion. *Astron. Astrophys.* **544**, A32 (2012).

[2] Johansen, A. et al. A pebble accretion model for the formation of the terrestrial planets in the Solar System. *Sci. Adv.* **7**, eabc0444 (2021). 33597233 10.1126/sciadv.abc0444 PMC7888959

[3] Morbidelli, A., Lunine, J. I., O’Brien, D. P., Raymond, S. N. & Walsh, K. J. Building terrestrial planets. *Annu. Rev. Earth Planet. Sci.* **40**, 251–275 (2012).

[4] Chambers, J. E. Late-stage planetary accretion including hit-and-run collisions and fragmentation. *Icarus* **224**, 43–56 (2013).

[5] Lissauer, J. J. et al. A closely packed system of low-mass, low-density planets transiting Kepler-11. *Nature* **470**, 53–58 (2011). 21293371 10.1038/nature09760

[6] Owen, J. E. & Mohanty, S. Habitability of terrestrial-mass planets in the HZ of M dwarfs. I. H/He-dominated atmospheres. *Mon. Not. R. Astron. Soc.* **459**, 4088-4108 (2016).

[7] Owen, J. E., Shaikhislamov, I. F., Lammer, H., Fossati, L. & Khodachenko, M. L. Hydrogen dominated atmospheres on terrestrial mass planets: evidence, origin and evolution. *Space Sci. Rev.* **216**, 129 (2020).

[8] Hogerheijde, M. R. in *Encyclopedia of Astrobiology* (eds Gargaud, M. et al.) 1357–1366 (Springer, 2011).

[9] Armitage, P. J. Dynamics of protoplanetary disks. *Annu. Rev. Astron. Astrophys.* **49**, 195–236 (2011).

[10] Hu, R., Seager, S. & Yung, Y. L. Helium atmospheres on warm Neptune- and sub-Neptune-sized exoplanets and applications to GJ 436b. *Astrophys. J.* **807**, 8 (2015).

[11] Cherubim, C., Wordsworth, R., Hu, R. & Shekolnik, E. Strong fractionation of deuterium and helium in sub-Neptune atmospheres along the radius valley. *Astrophys. J.* **967**, 139 (2024).

[12] Krishnamurthy, V. et al. Nondetection of helium in the upper atmospheres of TRAPPIST-1b, e, and f. *Astron. J.* **162**, 82 (2021).

[13] Van Looveren, G., Güdel, M., Boro, S. S. & Kislyakova, K. Airy worlds or barren rocks? On the survivability of secondary atmospheres around the TRAPPIST-1 planets. *Astron. Astrophys.* **683**, A153 (2024).

[14] Lammer, H. et al. Coronal mass ejection (CME) activity of low mass M stars as an important factor for the habitability of terrestrial exoplanets. II. CME-induced ion pick up of Earth-like exoplanets in close-in habitable zones. *Astrobiology* **7**, 185–207 (2007). 17407407 10.1089/ast.2006.0128

[15] Burgasser, A. J. & Mamajek, E. E. On the age of the TRAPPIST-1 system. *Astrophys. J.* **845**, 110 (2017).

[16] Peacock et al. Predicting the extreme ultraviolet radiation environment of exoplanets around low-mass stars: the TRAPPIST-1 system. *Astrophys. J.* **871**, 235 (2019).

[17] Fleming, D. P., Barnes, R., Luger, R. & VanderPlas, J. T. On the XUV luminosity evolution of TRAPPIST-1. *Astrophys. J.* **891**, 155 (2020).

[18] Duvvuri, G. M. et al. Reconstructing the extreme ultraviolet emission of cool dwarfs using differential emission measure polynomials. *Astrophys. J.* **913**, 40 (2021).

[19] Mamajek, E. E. Initial conditions of planet formation: lifetimes of primordial disks. *AIP Conf. Proc.* **1158**, 3–10 (2009).

[20] Richert, A. J. W. et al. Circumstellar disc lifetimes in numerous Galactic young stellar clusters. *Mon. Not. R. Astron. Soc.* **477**, 5191–5206 (2018).

[21] Heisch, K. E., Lada, E. A. & Lada, C. J. Disk frequencies and lifetimes in young clusters. *Astrophys. J.* **553**, 153–156 (2001).

[22] Siess, L., Dufour, E. & Forestini, M. An internet server for pre-main sequence tracks of low- and intermediate-mass stars. *Astron. Astrophys.* **358**, 593–599 (2000).

[23] Pfalzner, S., Dehghani, S. & Michel, A. Most planets might have more than 5 Myr of time to form. *Astrophys. J.* **939**, L10 (2022).

[24] Fedele, D., van den Ancker, M. E., Henning, T. H., Jayawardhana, R. & Oliveira, J. M. Timescale of mass accretion in pre-main-sequence stars. *Astron. Astrophys.* **510**, A72 (2010).

[25] Alexander, R., Pascucci, I., Andrews, S., Armitage, P. & Cieza, L. in *Protostars and Planets VI* (eds Beuther, H. et al.) 475–496 (Univ. Arizona Press, 2014).

[26] van Terwisga, S. E. & Hacar, A. Survey of Orion disks with ALMA (SODA). II. UV-driven disk mass loss in L1641 and L1647. *Astron. Astrophys.* **673**, L2 (2023).

[27] Pudritz, R. E. & Ray, T. P. The role of magnetic fields in protostellar outflows and star formation. *Front. Astron. Space Sci.* **6**, 54 (2019).

[28] Winter, A. J. & Haworth, T. J. The external photoevaporation of planet-forming discs. *Eur. Phys. J. Plus* **137**, 1132 (2022).

[29] Guarcello, M. G. et al. Dispersal timescale of protoplanetary disks in the low-metallicity young cluster Dolidze 25. *Astron. Astrophys.* **650**, A157 (2021).

[30] Rockcliffe, K. E. et al. The variable detection of atmospheric escape around the young, hot Neptune AU Mic b. *Astron. J.* **166**, 2023 (2023).

[31] Lundkvist M. S. et al. Hot super-Earths stripped by their host stars. *Nat. Commun.* **7**, 11201 (2016). 10.1038/ncomms11201 PMC4831017 27062914

[32] Fossati, L. et al. Aeronomical constraints to the minimum mass and maximum radius of hot low-mass planets. *Astron. Astrophys.* **598**, A90 (2017).

[33] Lammer, H., Brasser, R., Johansen, A., Scherf, M. & Leitzinger, M. Formation of Venus, Earth and Mars: constrained by isotopes. *Space Sci. Rev.* **2017**, 7 (2021).

[34] Lammer, H. et al. Measured atmospheric ^36^Ar/^38^Ar, ^20^Ne/^22^Ne, ^36^Ar/^22^Ne noble gas isotope and bulk K/U ratios constrain the early evolution of Venus and Earth. *Icarus* **339**, 11351 (2020).

[35] Lammer, H. et al. Origin and loss of nebula-captured hydrogen envelopes from ‘sub’- to ‘super-Earths’ in the habitable zone of Sun-like stars. *Mon. Not. R. Astron. Soc.* **439**, 3225–3238 (2014).

[36] Kubyshkina, D. et al. Grid of upper atmosphere models for 1–40 *M*_⊕_ planets: application to CoRoT-7 b and HD 219134 b,c. *Astron. Astrophys.* **619**, A151 (2018).

[37] Erkaev, N. V. et al. Modification of the radioactive heat budget of Earth-like exoplanets by the loss of primordial atmospheres. *Mon. Not. R. Astron. Soc.* **518**, 3703–3721 (2023).

[38] Owen, J. E. & Jackson, A. P. Planetary evaporation by UV & X-ray radiation: basic hydrodynamics. *Mon. Not. R. Astron. Soc.* **425**, 2931–2947 (2012).

[39] Kubyshkina, D. et al. Young planets under extreme UV (EUV) irradiation. I. Upper atmosphere modelling of the young exoplanet K2-33b. *Astron. Astrophys.* **612**, A25 (2018).

[40] Kubyskina, D., Fossati, L. & Erkaev, N. V. Precise photoionization treatment and hydrodynamic effects in atmospheric modelling of warm and hot Neptunes. *Astron. Astrophys.* **684**, A26 (2024).

[41] Guo, J. H. Characterization of the regimes of hydrodynamic escape from low-mass exoplanets. *Nat. Astron.* **8**, 920–928 (2024).

[42] García Muñoz, A. Heating and ionization by non-thermal electrons in the upper atmospheres of water-rich exoplanets. *Astron. Astrophys.* **672**, A77 (2023).

[43] Guo, J. H. & Ben-Jaffel, L. The influence of the extreme ultraviolet spectral energy distribution on the structure and composition of the upper atmosphere of exoplanets. *Astrophys. J.* **818**, 107 (2016).

[44] Kubyshkina, D. et al. Close-in sub-Neptune reveal the past rotation history of their host stars: atmospheric evolution of planets in the HD 3167 and K2-32 planetary systems. *Astrophys. J.* **879**, 26 (2019).

[45] Owen, J. E. & Wu, Y. Atmospheres of low-mass planets: the ‘boil-off’. *Astrophys. J.* **817**, 107 (2016).

[46] Ikoma, M., Nakazawa, K. & Hiroyuki, E. Formation of giant planets: dependences on core accretion rate and grain opacity. *Astrophys. J.* **537**, 1013–1025 (2000).

[47] Lee, E. J. The boundary between gas-rich and gas-poor planets. *Astrophys. J.* **878**, 36 (2019).

[48] Inamdar, N. K. & Schlichting, H. E. Stealing the gas: giant impacts and the large diversity in exoplanet densities. *Astrophys. J. Lett.* **817**, L13 (2016).

[49] Schlichting, H. E., Sari, R. & Yalinewich, A. Atmospheric mass loss during planet formation: the importance of planetesimal impacts. *Icarus* **247**, 81–94 (2015).

[50] Tu, L., Johnstone, C. P., Güdel, M. & Lammer, H. The extreme ultraviolet and X-ray Sun in time: high-energy evolutionary tracks of a solar-like star. *Astron. Astrophys.* **577**, L3 (2015).

[51] Zhang, M., Knutson, H. A., Wang, L., Dai, F. & Barragán, O. Escaping helium from TOI 560.01, a young mini-Neptune. *Astrophys. J.* **163**, 67 (2022).

[52] Allart, R. et al. Spectrally resolved helium absorption from the extended atmosphere of a warm Neptune-mass exoplanet. *Science* **362**, 1384–1387 (2018). 30523080 10.1126/science.aat5879

[53] Nortmann, L. et al. Ground-based detection of an extended helium atmosphere in the Saturn-mass exoplanet WASP-69b. *Science* **362**, 1388–1391 (2018). 30523081 10.1126/science.aat5348

[54] Ferland, G. J. et al. The 2017 release of Cloudy. *Rev. Mex. Astron. Astrofís.* **53**, 385–438 (2017).

[55] Chatzikos, M. et al. The 2023 release of Cloudy. *Rev. Mex. Astron. Astrofís.* **59**, 327–343 (2023).

[56] Fossati, L. et al. Non-local thermodynamic equilibrium effects determine the upper atmospheric temperature structure of the ultra-hot Jupiter KELT-9b. *Astron. Astrophys*. **653**, A52 (2023).

[57] Young, M. E. et al. Non-local thermodynamic equilibrium transmission spectrum modelling of HD 209458b. *Astron. Astrophys.* **641**, A47 (2020).

[58] Orell-Miquel, J. et al. The MOPYS project: A survey of 70 planets in search of extended He i and H atmospheres. No evidence of enhanced evaporation in young planets. *Astron. Astrophys.* **689**, A179 (2024).

[59] Masson, A. et al. Probing atmospheric escape through metastable He i triplet lines in 15 exoplanets observed with SPIRou. *Astron. Astrophys.* **688**, A179 (2024).

[60] Guilluy, G. et al. The GAPS programme at TNG. LIV. A He i survey of close-in giant planets hosted by M-K dwarf stars with GIANO-B. *Astron. Astrophys.* **686**, A83 (2024).

[61] Scott, I. Stay out of that ballon! *Inj. Prev.* **12**, 322 (2006).

[62] Scherf, M. et al. Simulation data for Lammer, H., Scherf, M., et al. Earth-mass planets with He atmospheres in the habitable zone of Sun-like stars, *Nature Astronomy*, accepted, 2025. *Figshare*10.6084/m9.figshare.28533617 (2025). 10.1038/s41550-025-02550-6 PMC12274132 40687773

